# Three Years of Experience Using a Diode Laser in the Management of Common Anal Benign Pathologies: A Retrospective Study

**DOI:** 10.7759/cureus.93619

**Published:** 2025-09-30

**Authors:** Mohamed Abdelhalim, Ahmed H. Amer, Hamdy Abdel-Hady, Mostafa M Elsheikh

**Affiliations:** 1 General Surgery, Tanta University, Tanta, EGY; 2 General Surgery, Tanta University Hospitals, Tanta, EGY

**Keywords:** chronic fissure, diode laser, filac, hemorrhoidoplasty, hemorrhoids, perianal fistula

## Abstract

Background

The management of anorectal conditions continues to evolve with newer techniques for colorectal surgeons. Recently, diode laser technology has gained attention in proctology as a less invasive alternative to traditional surgery. The mechanism involves tissue shrinkage and degeneration and depends on the power of the laser and the duration of exposure. This method is associated with reductions in postoperative pain, bleeding, discharge, and hospital stays.

Patients and methods

This retrospective study involved 456 patients with hemorrhoids, chronic anal fissure, and perianal fistula. They were divided into three groups. Group 1 (156 patients) had hemorrhoids treated with laser hemorrhoidoplasty (LHP). Group 2 (144 patients) had chronic anal fissures and underwent closed laser lateral internal sphincterotomy. Group 3 (156 patients) had perianal fistulas managed either by fistula laser closure (FILAC) or laser fistulotomy. All of the procedures were performed at the Gastrointestinal Surgery Unit in the General Surgery Department at Tanta University Hospitals in Egypt over a three-year period (February 2021 to February 2024).

Results

In Group 1, LHP resulted in minimal postoperative pain (as measured by the visual analogue score), negligible bleeding (with no cases requiring intervention), brief hospital stays, and a low recurrence rate (one case). Postoperative edema was significantly associated with grade 4 hemorrhoids. Group 2 experienced no major complications, though 30 patients reported postoperative itching, which resolved with topical treatment. In Group 3, the success rate for FILAC was 73.9%, and the overall success rate was 76.9%. Recurrence correlated significantly with posteriorly located intersphincteric fistulas and fistulous tracts wider than 7 mm.

Conclusion

Diode laser treatment is a safe and minimally invasive option for managing hemorrhoids, chronic anal fissures, and perianal fistulas that results in favorable outcomes and minimal complications.

## Introduction

Anorectal disorders encompass a range of conditions affecting the junction between the rectum and the anal canal. Although these conditions are common in the general population, their true incidence is likely underreported because many individuals do not seek medical care [1]. The most frequent benign anorectal diseases include hemorrhoids, anal fissures, and anal fistulas, all of which can substantially impair a patient's quality of life [2]. Hemorrhoidal disease is characterized by the congestion of blood vessels around the anal canal. Typical symptoms include rectal bleeding, prolapse, itching, fecal soiling, and psychological distress [2]. An anal fissure is a small tear or crack in the anal mucosa, usually extending from the dentate line to the anal verge. Fissures that persist beyond four to eight weeks are classified as chronic [3]. Anal fistulas are abnormal epithelialized tracts connecting the anal canal to the perianal skin. They are categorized by their anatomical course as either intersphincteric, transsphincteric, suprasphincteric, or extrasphincteric [4].

Surgical intervention has traditionally been regarded as the definitive and, often, more effective treatment for these conditions compared with conservative medical therapies [5]. However, conventional surgical procedures such as hemorrhoidectomy for hemorrhoids, lateral internal sphincterotomy for chronic fissures, and fistulectomy for fistulas can result in varying degrees of postoperative pain, bleeding, delayed recovery, and recurrence, outcomes that are influenced by the choice of technique and the surgeon’s expertise [6].

Among emerging treatments, medical lasers-including diode, carbon dioxide, argon, and Nd:YAG types-induce tissue shrinkage and degeneration at controlled depths depending on the intensity and duration of exposure. Recently, diode laser therapy has gained attention as a minimally invasive and nearly painless alternative to conventional surgery that offers benefits such as reduced postoperative pain, minimal bleeding, and faster recovery, thus enabling patients to resume daily activities sooner [7].

## Materials and methods

This retrospective study included 456 patients who presented with common anal afflictions (hemorrhoids, chronic fissures, and fistulas). They were admitted to the General Surgery Department at Tanta University Hospitals in Egypt from February 2021 to February 2024. We divided these patients into three groups. Group 1 included 156 patients with hemorrhoids, Group 2 included 144 patients with chronic anal fissures, and Group 3 included 156 patients with perianal fistulas. The hospital record system for inpatients was used for data collection.

The inclusion criteria included being more than 18 years of age and having second-, third-, or fourth-degree hemorrhoids, chronic anal fissures, or perianal fistulas. Patients with acute anal fissures, acutely inflamed thrombosed hemorrhoids, and/or inflammatory bowel disease (IBD) affecting the rectum or anus were excluded from the study.

Preoperative assessment

All of the patients were subjected to full history-taking, a general examination, a digital rectal examination to determine the degree of hemorrhoids, the position of anal fissures, or the internal and external openings of perianal fistulas, routine laboratory investigations, colonoscopy to exclude cancer of the colon or rectum in patients over 50 years old, endo-anal ultrasound for cases of perianal fistula, and MRI for cases of suspected complex perianal fistula.

 Written informed consent was obtained from all patients included in the study. Patients were informed of the purpose, procedures, potential risks, and benefits of the research. They were assured of the confidentiality of their responses and their right to withdraw from the study at any time without penalty. The study was reviewed and approved by the Research Ethics Committee, Faculty of Medicine, Tanta University (approval number: 34385/1/21). All procedures performed in this study involving human participants were in accordance with the ethical standards of the institutional research committee and with the 1964 Helsinki declaration and its later amendments. Informed consent was obtained from all individual participants included in the study.

Operative technique

Group 1 (Hemorrhoids)

Laser hemorrhoidoplasty (LHP) was defined by three positions. Position A was the coagulation of the feeding vessel of the hemorrhoid by introducing the bare fibers through the anus and delivering approximately 80 joules without touching the mucosa to avoid injuring it. Position B involved using the laser probe to make a skin micro-incision of 3 mm, approximately 0.5 cm from the anal verge at the base of each hemorrhoid in the subcutaneous plane. The probe was driven through the incision in the submucosal tissue until it reached the area underneath the rectal mucosa. Effective pulses of approximately 100 joules from a laser generator were fired using bare fibers under 8 watts at a wavelength of 1470 nm. Position C was then achieved by delivering another 80-100 joules to the cushion to achieve shrinkage of the hemorrhoids. Approximately 250-300 joules in total were delivered to each hemorrhoid. Ice packs were placed inside the anus to produce a cooling effect and avoid postoperative itching caused by the heat of the laser. In cases of fourth-degree hemorrhoids with a large external component, mucopexy was performed to augment shrinkage.

Group 2 (Chronic Anal Fissure)

Laser lateral internal sphincterotomy was performed using bare fibers by the closed method. Palpation of the internal sphincter using this method was followed by the introduction of the laser probe through the skin toward the internal sphincter and the delivery of 70-100 joules. Next, the surgeons used the left index finger to separate the internal sphincter with minimal pressure to detect the defect in the internal anal sphincter. As with Group 1, ice packs were placed inside the anus to produce a cooling effect and avoid postoperative itching caused by the heat of the laser.

Group 3 (Perianal Fistula)

Fistula catheterization by probe was performed to identify the internal opening. In some cases, an injection of hydrogen peroxide was necessary to detect this opening. The internal orifice was closed using either a figure-eight Vicryl 3-0 suture (Ethicon, Inc., Raritan, NJ, USA) or a mucosal advancement flap. In cases in which the internal opening was not present or the track was blind-ended, only fistula laser closure (FILAC) was performed. The laser radial fibers were introduced from the internal to the external orifices of the fistula. Laser closure of the fistulous track was done with simultaneous destruction and sealing of the track. One hundred joules were delivered for each 1 cm of the track using radial fibers of 1470 nm in wavelength and 10 watts of energy. Coagulation of the external orifice was performed at the end of the procedure. In cases in which a fistula was associated with discharge, proper drainage of the external orifice was achieved, and then curettage was performed, followed by washing with hydrogen peroxide and saline. In cases of posterior intersphincteric fistulas or tracks more than 7 mm in width, laser fistulotomy was performed using bare fibers of 1470 nm in wavelength and 10 watts of energy. Ligation of the intersphincteric fistula track combined with FILAC was performed in some cases of complex perianal fistula. Ligation of the intersphincteric fistula tract (LIFT) was performed proximally, followed by FILAC for coagulation of the track distally.

Follow-up consisted of assessing postoperative pain using the visual analogue scale (VAS) as well as the need for analgesics, postoperative bleeding (either spontaneous or post-defecation), edema, and sero-mucous discharge in the first 24 hours. Patients were discharged 24 hours after surgery or on the same day when there were no complications and their pain was less than 5 on the VAS score. Follow-up continued weekly for one month, monthly for six months, and once yearly.

Statistical analysis

The data were collected, tabulated into a computer, and analyzed using IBM SPSS Statistics for Windows, version 20 (IBM Corp., Armonk, NY, USA). The qualitative data were described as numbers and percentages. The Shapiro-Wilk test was used to verify the normality of the distribution. The quantitative data were described using minimum and maximum range, mean, standard deviation, median, and interquartile range. The significance of the results thus obtained was assessed at the 5% level. The Chi-square test was used to compare the categorical variables across the groups. Fisher’s exact test or the Monte Carlo correction for the Chi-square was used when more than 20% of the cells had an expected count of less than 5, and Student’s t-test was used to compare the normally distributed quantitative variables among the three study groups.

## Results

Group 1 (hemorrhoids)

The age of the 156 patients who underwent LHP ranged from 22 to 76 years. Of these patients, 102 were male (65.4%), and 54 were female (34.6%). Sixty (38.5%) suffered from grade 2 hemorrhoids, 54 (34.6%) from grade 3 hemorrhoids, and 42 (26.9%) from grade 4 hemorrhoids. The operative time ranged from six to 23 minutes, with a mean of 13.19 ± 5.04 SD. Partial shrinkage of the patients’ hemorrhoids occurred immediately postoperatively, and complete shrinkage occurred after one month.

Postoperative pain was evaluated using the VAS score. In the first 6 hours after surgery, these scores ranged from 4 to 9, with a mean of 5.81 ± 1.23 SD. After 12 hours, the scores ranged from 1 to 7, with a mean of 4.19 ± 1.70 SD. After 24 hours, they ranged from 0 to 7, with a mean of 2.73 ± 2.07 SD. Three days after surgery, they ranged from 0 to 3, with a mean of 0.12 ± 0.59 SD. The patients’ VAS scores were 0 after one week, two weeks, one month, two months, and six months of follow-up.

Postoperative bleeding was present in the form of spontaneous post-defecation spotting, but there was no serious bleeding requiring hemostasis. Bleeding occurred in 18 patients (11.5%) after 24 hours and in six patients (3.8%) after one week, two weeks, one month, two months, and six months, which was considered a recurrence. There was no postoperative discharge in any cases after 24 hours, but discharge was observed in six cases (3.8%) after one week in the form of infection and was treated conservatively with antibiotics such as ceftriaxone or levofloxacin. Postoperative edema was present in 36 patients (23.1%) and was treated conservatively with local and systemic anti-inflammatory medications (Table 1).

**Table 1:** Distribution of complications in Group 1 (n = 156)

Complication	Number of patients	Percent of patients
Postoperative edema	36	23.1
Infection	12	7.7
Recurrence	6	3.8
Itching	18	11.5
Incontinence	0	0.0
Stenosis	0	0.0

There was a significant correlation between the grade of hemorrhoid and postoperative edema (p < 0.001), as the 36 patients who developed edema had grade 4 hemorrhoids. A correlation was also observed between grade 4 hemorrhoids and the development of other postoperative complications, but it was not significant (p = 0.066 for infection; p = 0.260 for recurrence) (Table 2). Itching occurred in 18 patients (11.5%), mainly because of the heat generated by the laser probe, and was treated with local soothing agents. Infections occurred in 12 patients (7.7%) and were treated with antibiotics.

**Table 2:** Relationship between hemorrhoid grade and complications in Group 1 (n = 156) MC: Monte Carlo correlation; No.: number

Complications	Grade						Chi-square	^MC^P-value
	II (n = 10)		III (n = 9)		IV (n = 7)			
	No.	%	No.	%	No.	%		
Postoperative edema	0	0.0	0	0.0	36	85.7	17.396	<0.001
Infection	0	0.0	0	0.0	12	28.6	3.905	0.066
Recurrence	0	0.0	0	0.0	6	14.3	2.356	0.260
Itching	0	0.0	12	22.2	6	14.3	2.368	0.339
Incontinence	0	0.0	0	0.0	0	0.0	-	-
Stenosis	0	0.0	0	0.0	0	0.0	-	-

There was no incontinence or stenosis in any of the 156 patients. Recurrence occurred in six patients (3.8%), who then underwent conventional hemorrhoidectomy after six months of follow-up (Table 1). The length of the patients’ hospital stays ranged from one to two days, with a mean of 1.12 ± 0.33 SD. They returned to normal activities within two to seven days after surgery (mean 3.73 ± 1.34 SD).

Group 2 (chronic anal fissure)

The 144 patients who underwent laser closed lateral internal sphincterotomy using a diode laser with bare fibers and a wavelength of 1470 nm ranged in age from 20 to 62 years. Of these patients, 48 were male (33.3%), and 96 were female (66.7%). The operative time ranged from three to 12 minutes, with a mean of 6.54 ± 2.45 SD. Postoperative pain was evaluated using the VAS score. After six hours, the patients’ VAS scores ranged from 3 to 8, with a mean of 6.21 ± 1.28 SD. After 12 hours, their scores ranged from 1 to 7, with a mean of 3.83 ± 1.61 SD. After 24 hours, their scores ranged from 0 to 6, with a mean of 1.58 ± 1.32 SD. After three days, their scores ranged from 0 to 1, with a mean of 0.04 ± 0.20 SD. The patients’ VAS scores were 0 after one week, two weeks, one month, two months, and six months of follow-up.

Regarding postoperative bleeding, six of the patients in this group (4.2%) developed bleeding during the first 24 hours after surgery in the form of post-defecation spotting, but none experienced serious bleeding requiring surgical hemostasis. None of the patients in this group experienced bleeding from three days after surgery to the six-month follow-up. None of the patients in this group experienced postoperative discharge in the first 24 hours after surgery. Twelve of them (8.3%) developed a serous discharge after three days that continued for one week, but the discharge ceased within two weeks and was not observed at the six-month follow-up. No cases of incontinence, stenosis, recurrence, postoperative edema, or infection were recorded among the members of this group. Thirty of these patients (20.8%) reported itching, which was treated with local soothing agents. Their hospital stays ranged from 1 to 2 days, and they returned to normal activities within two to seven days.

Group 3 (perianal fistula)

The 156 consecutive patients who underwent laser treatment for perianal fistula ranged in age from 17 to 72 years, with a mean of 42.58 ± 12.37 SD. Of these patients, 102 (65.4%) were male and 54 (34.6%) were female. The position of the fistula in 78 of the patients in this group (50%) was anterior, while the position was posterior in 72 of them (46.2%), and six (3.8%) had both anterior and posterior fistulas. The fistulas in 18 of these cases (11.5%) were recurrent. Regarding the type of the fistula, 60 cases (76.9%) had intersphincteric tracks, 30 cases (19.2%) had transsphincteric tracks, and six cases (3.8%) had suprasphincteric tracks. The affection of the external sphincter was present in 36 patients (23.1%) and was documented by endo-anal ultrasound in routine imaging for all of the cases of perianal fistula. Eighteen cases (11.5%) were associated with discharge or abscess cavity, and 60 patients (38.5%) had a history of previous anorectal surgery (hemorrhoids, anal fissure, anal fistula, and/or perianal abscess).

The caliber of the track ranged from 3 to 11 mm, with a mean of 6.36 mm. One hundred and twenty-six patients (80.2%) had a caliber of ≤ 7 mm, while 30 patients (19.2%) had a caliber of > 7 mm. The length of the tracks ranged from 2.3 to 8.0 cm, with a mean of 3.75 ± 1.49 SD. One hundred and thirty-two patients (88.5%) had a single fistulous track, 12 patients (7.7%) had two tracks, and six patients had four tracks (3.8%), which were biopsied to exclude IBD. Thus, the total number of tracks was 186. The operative time ranged from five to 23 minutes, with a mean of 11.73 ± 4.90 SD. One hundred and thirty-eight patients (88.5%) underwent FILAC using a diode laser at a wavelength of 1470 nm with radial fibers, and 18 patients (11.5%) underwent fistulotomy using a diode laser at a wavelength of 1470 nm with bare fibers. Twelve patients (7.7%) underwent FILAC combined with LIFT, and 18 patients (11.5%) underwent FILAC with excision of the external opening for proper drainage. The patients’ hospital stays ranged from one to three days, and they returned to normal activities within three to 12 days.

Postoperative pain was evaluated using the VAS score. Six hours after the surgery, the VAS scores ranged from 4 to 8, with a mean of 6.27 ± 1.28 SD. Twelve hours after surgery, the range was from 2 to 8, with a mean of 4.35 ± 2.08. After 24 hours, the VAS scores continued to decrease, ranging from 0 to 8, with a mean of 2.54 ± 2.10 SD. After three days, the VAS scores ranged from 0 to 2, with a mean of 0.31 ± 0.62 SD. After one week, the range was from 0 to 1, with a mean of 0.04 ± 0.20 SD. The scores then reached 0 after two weeks and remained 0 at one month, two months, and six months of follow-up. There was no postoperative bleeding in any of the 156 cases during the first 24 hours or up to six months of follow-up. No postoperative discharge was recorded in the first 24 hours, but discharge was recorded on the third day of follow-up in 126 patients (80.8%), in 90 patients (57.7%) after one week, in 54 patients (34.6%) after two weeks, and in 36 patients (23.1) after one month, two months, six months, and up to one year of follow-up, which was considered recurrence. Notably, the 24 patients who suffered from recurrence had posterior intersphincteric fistulas and underwent FILAC, with a caliber track of more than 7 mm in width and variable length. Accordingly, we performed laser fistulotomy on the other patients who suffered from posterior intersphincteric fistulas.

During the follow-up, which was up to six months, postoperative perianal edema occurred in 12 patients (7.7%) and was treated with anti-inflammatory medications. Infections developed in six cases (3.8%) in the recurrence group and were treated with antibiotics followed by open fistulotomy. No incontinence or stenosis occurred in any of the cases. Itching developed in 12 cases (7.7%) and was treated with local soothing agents. The success rate of the cases that underwent FILAC was 73.9% and 100% for the cases that underwent laser fistulotomy. Recurrence occurred in 36 patients (23.1%), who were treated with lay-open fistulotomy.

There was a significant correlation between the posterior position of the fistula and recurrence (p < 0.001), while other complications, such as edema, itching, and infection, did not correlate significantly with the position of the fistula (Table 3).

**Table 3:** Relationship between the position of fistulas and complications in FILAC cases (n = 46) MC: Monte Carlo correlation; No.: number; FILAC: fistula laser closure

Complication	Position of fistula						Chi-square	^MC^P-value
	Anterior (n = 26)		Posterior (n = 18)		Anterior & posterior (n = 2)			
	No.	%	No.	%	No.	%		
Postoperative edema								
No	26	100.0	14	77.8	2	100.0	3.664	0.221
Yes	0	0.0	4	22.2	0	0.0		
Infection								
No	26	100.0	16	88.9	2	100.0	2.934	0.442
Yes	0	0.0	2	11.1	0	0.0		
Recurrence								
No	26	100.0	6	33.3	2	100.0	12.173	0.001
Yes	0	0.0	12	66.7	0	0.0		
Itching								
No	26	100.0	14	77.8	2	100.0	3.664	0.221
Yes	0	0.0	4	22.2	0	0.0		
Incontinence								
No	26	100.0	18	100.0	2	100.0	–	–
Yes	0	0.0	0	0.0	0	0.0		

Notably, recurrence was significant in the patients who had a caliber track of > 7 mm and underwent FILAC. Thus, 24 of the 30 cases (80%) with a caliber track of > 7 mm suffered from recurrence, while only 12 of the 108 cases (11.1%) with a caliber of ≤ 7 mm suffered from recurrence (Table 4).

**Table 4:** Relationship between the caliber of the track and complications in FILAC cases (n = 46) No.: number; SD: standard deviation; FE: Fisher’s exact; FILAC: fistula laser closure

Complication	Caliber of the track (mm)						
	N	Mean ± SD	Median (Min. – Max.)	≤ 7 (n = 36)		> 7 (n = 10)	
				No.	%	No.	%
Postoperative edema							
No	42	6.40 ± 1.78	6.0 (3.0–11.0)	32	88.9	10	100.0
Yes	4	6.50 ± 0.71	6.50 (6.0–7.0)	4	11.1	0	0.0
Test of significance (p)	–	t=0.077; p=0.939		χ^2^=0.608; ^FE^p=1.000			
Infection							
No	44	6.43 ± 1.74	6.45 (3.0–11.0)	34	94.4	10	100.0
Yes	2	6.0		2	5.6	0	0.0
Test of significance (p)	–	–		χ^2^=0.290; ^FE^p=1.000			
Recurrence							
No	34	5.79 ± 1.23	6.0 (3.0–7.50)	32	88.9	2	20.0
Yes	12	8.17 ± 1.72	8.0 (6.0–11.0)	4	11.1	8	80.0
Test of significance (p)	–	t=3.678; p=0.001		χ^2^=9.631; ^FE^p=0.008			
Itching							
No	42	6.35 ± 1.75	6.0 (3.0–11.0)	34	94.4	8	80.0
Yes	4	7.0 ± 1.41	7.0 (6.0–8.0)	2	5.6	2	20.0
Test of significance (p)	–	t=0.504; p=0.619		χ^2^=1.028; ^FE^p=0.395			
Incontinence							
No	46	6.41 ± 1.71	6.0 (3.0–11.0)	36	100.0	10	100.0
Yes	0	–		0	0.0	0	0.0
Test of significance (p)	–	–		–			

Other complications, such as edema, itching, and the formation of abscesses, did not correlate significantly with the caliber of the track. There was also no significant correlation between the type of fistula and postoperative complications, for all of the recurrent cases were of the intersphincteric type. Further, there was no significant correlation between the length of the fistulous track and postoperative complications. Notably, there was a significant correlation between recurrence and the cases with both posterior fistula and a track caliber of > 7 mm (p = 0.002) (Table 5).

**Table 5:** Relationship between posterior fistula, the caliber of the track > 7 mm, and recurrence in FILAC cases (n = 46) No.: number; FE: Fisher’s exact; FILAC: fistula laser closure

Recurrence	Posterior fistula and caliber of the track > 7 mm				Chi-square	^FE^p
	No (n = 38)		Yes (n = 8)			
	No.	%	No.	%		
No	34	89.5	0	0.0	13.719	0.002
Yes	4	10.5	8	100.0		

## Discussion

Treatment of anorectal diseases has always been challenging because there are many treatment options. Weyand et al. suggested that these options create confusion about the best modality for treatment, which remains controversial, especially when experienced colorectal surgeons are required for advanced and new techniques or if the institution lacks facilities for certain procedures [8]. In our study, we used a diode laser to treat common anal surgical lesions, specifically, hemorrhoids, chronic fissures, and fistulas. Of the 456 patients included in the study, 156 suffered from hemorrhoids, 144 suffered from chronic anal fissures, and 156 suffered from perianal fistulas.

Hemorrhoids

We used a diode laser at a wavelength of 1470 nm and a power of 8 watts. The total joules delivered ranged from 250 to 350 for each hemorrhoid. Mucopexy was associated with LHP in cases with grade 4 hemorrhoids. The follow-up period ranged from one year to three years. Regarding the wavelength, Maloku et al. and Naderan et al. used a diode laser at a wavelength of 980 nm, while Brusciano et al., Weyand et al., and Poskus et al. used a diode laser at a wavelength of 1470 nm, as in our study [2,5,7-9].

Maloku et al. compared LHP with open hemorrhoidectomy for the treatment of third- and fourth-degree hemorrhoids and concluded that patients who underwent the former procedure experienced less pain, their operative times were shorter, and they returned to normal activities more quickly than those who underwent the latter procedure [2]. Weyand et al. recommended that the energy delivered during LHP should be reduced to a minimum and not exceed 500 joules in order to avoid complications such as perianal edema and thrombosis, especially in the external component of the hemorrhoid [8]. These researchers also performed associated mucopexy in indicated cases of grade 4 hemorrhoids to augment proper shrinkage, similar to our study. Giamundo et al. also performed mucopexy, in this case, using the hemorrhoidal laser procedure (HeLP) to treat third-degree hemorrhoids [10]. The procedure showed promising results regarding postoperative pain and bleeding, and return to normal activities. For the treatment of second- and third-degree hemorrhoids, Poskus et al. compared LHP with sutured mucopexy and open hemorrhoidectomy and concluded that LHP was associated with less postoperative pain, less operative time, and more rapid return to normal activities than the other two procedures and that patients rated LHP higher than those procedures [9]. Plapler et al. reported that open hemorrhoidectomy was more effective than LHP with respect to recurrence and that LHP was more expensive than the other procedures [11]. Brusciano et al. performed LHP on 50 patients with symptomatic hemorrhoids, found results similar to ours: the procedure was associated with minimal discomfort or postoperative pain, with a mean VAS score of 2 (range 0-3), while, over subsequent days, the scores decreased to 0, and the patients experienced relatively short hospital stays and quickly returned to normal activities, with no recurrence [7]. These researchers also found that the use of a wavelength of 1470 nm, penetrating only 2 mm, was associated with optimum shrinkage of hemorrhoidal tissue and was selectively better adsorbed by hemoglobin than Nd:YAG laser treatment. Naderan et al. also reported that LHP had better outcomes than Milligan-Morgan hemorrhoidectomy in terms of operative time, postoperative pain, and bleeding, but was more expensive [7]. Thrombosis of external hemorrhoids occurred in two of 30 patients because the external portion lost its venous drainage.

Chronic anal fissure

Lasers are less commonly used to treat chronic anal fissures than hemorrhoids or perianal fistulas. Esfahani et al. used a carbon dioxide laser as a novel method to treat anal fissures and reported that this new laser-based surgical approach is simple, safe, and effective and can be performed with local anesthesia in an outpatient clinic with minimal postoperative morbidity [12].

Alam et al. likewise performed LHP and closed laser LIS on 120 patients complaining of anal fissure and hemorrhoids [13]. The most frequently reported complications were bleeding, in 63 cases (52.5%), pain, in 55 cases (45.8%), and itching, in 21 cases (22%). These researchers concluded that diode laser surgery is a minimally invasive procedure that can even be performed as a day-care procedure and offers several advantages over traditional surgery. Thus, the procedure effectively treated hemorrhoids and anal fissures, improving symptoms and reducing postoperative pain.

Perianal fistula

The treatment of perianal fistulas has always been challenging despite the presence of many treatment options. Mitalas et al. reported that the most common causes of failure or recurrence of a fistula are missed internal opening, missed side tracks, and improper drainage [14]. Sygut et al. reported that incontinence was the most feared complication in the treatment of complex anal fistula with affection of the sphincter, especially with respect to conventional surgery [15]. Wilhelm A. was the first to use radial-emitting diode laser fibers to treat perianal fistulas in a 2011 pilot study involving 11 patients suffering from the condition [16]. Initially, all of the patients underwent drainage of perianal abscesses and a seton for localization of the internal opening and reduction of the local infection. These researchers used a diode laser at a wavelength of 1470 nm because it had the optimal adsorption curve in water, with a defined penetration depth limited to 2-3 mm. This approach proved superior to the use of wavelengths such as 980 nm, which was associated with vascular injury in the laser treatment of varicose veins. Wilhelm A. closed the internal opening with a Vicryl 2-0 suture together with the advancement flap technique, introducing the laser probe through the external opening at a power of 13 watts with continuous retraction of the probe at a rate of three seconds per centimeter [16]. They achieved a success rate of 81.8% and argued that sealing perianal fistulas using the FILAC method was a promising novel approach for saving the sphincter with minimal complications, but that further studies were needed to assess it.

In our study, 156 patients underwent laser treatment for perianal fistulas. The FILAC method was used for 138 of them, and laser fistulotomy for the other 18. We used a diode laser at a wavelength of 1470 nm and a power of 10 watts with radial fibers, except for laser fistulotomies, in which case we used bare fibers. We did not apply a seton, as Wilhelm A. did; so, in cases of associated abscess or infection, wide excision of the external opening and drainage of the abscess cavity was performed [16]. The catheterization of the fistula was followed by the introduction of the laser probe from the external to the internal opening so that FILAC would start from the latter. Energy was applied at a rate of 100 joules per centimeter. We considered the sticky sensation that was perceived each time the probe was retracted a positive indication of sealing and fibrosis of the track. The internal opening was closed with a Vicryl 3-0 suture. The endo-anal mucosal flap technique was performed in two cases to close the internal opening. In two other cases, the LIFT technique was accompanied by the FILAC technique. We recorded a success rate of 73.9% among the patients who underwent FILAC.

Giamundo et al. performed FILAC on 35 patients with transsphincteric or anterior intersphincteric fistulas but excluded superficial fistulas that could be treated by fistulotomy [4]. Of these patients, 16 had previous seton placement. Both 1470 nm and 980 nm wavelengths were used, the internal opening was left without any means of closure, and excessive debridement of the track was avoided because the researchers were concerned that unnecessary widening of the track would increase the possibility of recurrence. They reported a success rate of 71.4% and results that were similar to ours regarding operative time and postoperative pain, bleeding, and incontinence. These researchers also found that the 1470 nm wavelength had a better sealing effect and caused less thermal damage than the 980 nm wavelength. In addition, the success rate was higher among the patients who had previous seton placement because the seton localized the track and minimized its caliber. They concluded that FILAC is a minimally invasive sphincter-saving procedure with minimal postoperative complications, favorable results, and a short learning curve, but described the technique as blind in that it does not detect any side branches of a fistula, which could cause recurrence. They also described the high cost as a disadvantage.

Complete shrinkage of the pathology is mandatory to increase the success rate, as Doganci et al. reported [17]. Öztürk and Gülcü reported a high success rate of 82% when they performed FILAC on 50 patients with transsphincteric or intersphincteric fistulas [18]. Seton placement was used for three to four weeks before FILAC in cases of associated abscesses. These researchers suggested that debridement of the track with a cytology brush was important to avoid widening it and that hemoglobin is the ideal protein for sealing it. By contrast, in our study, a simple curette was used for debridement of the track, 100 joules per cm were delivered, and the internal opening was closed. Öztürk and Gülcü reported that to-and-fro movement toward the internal opening while performing FILAC was essential to enhance sealing of the track, describing this procedure as effective for treating anal fistulas, especially those involving the external sphincter [18]. Giamundo et al. reported a 71.1% success rate over 30 months of follow-up among 45 patients who underwent FILAC using a diode laser at a wavelength of 1470 nm [19]. They found that patients who had been treated with a loose seton eight weeks before the laser treatment had a higher rate of healing (79%) than patients without a seton (62%). They also found that a better healing rate was achieved when the length of the track was ≥ 4 cm and that fistulas of ≤ 2 cm in length should not receive FILAC treatment.

We recommend avoiding the to-and-fro movement performed by Öztürk and Gülcü because it could hamper the healing effect of the laser [18]. We did not use this kind of movement because the newly formed tissue in the lumen of the fistula was still incomplete and vulnerable during the procedure and could easily have been perforated by the laser probe if it were pushed back into the treated tract, as Wilhelm et al. recommended [20]. The first long follow-up period for patients who underwent FILAC was five years, with Wilhelm et al. reporting a success rate of 64.1% when the procedure was performed on 117 patients suffering from perianal fistulas. These researchers suggested that the placement of a seton facilitates the introduction of the laser probe from the external to the internal opening and that the laser probe should pass through the internal fistula opening to reach the “turning point” of the fistula track and obliterate the intersphincteric component. For our recurrent patients, we performed fistulotomies after six months, while Wilhelm et al. performed re-FILAC, fistulotomy, or fistulectomy [20] . They reported a secondary success rate of 88% and proposed that FILAC be considered a sensible first line of treatment for perianal fistulas and that a failed FILAC allows for an easier fistulectomy.

Carvalho et al. excised the external opening to improve wound drainage and did not use a seton, similar to the approach in our study [21]. However, instead of closing the internal opening with sutures, they closed it with a myo-mucosal flap. Lauretta et al. argued that the only significant factor in the treatment of fistulas by FILAC is the length of the track, but this factor was not significant in our series: the success rate for fistulas with a track length of < 3 cm was 58.3%, while it was 16.6% for fistulas with a track length of > 3 cm [22]. By contrast, Giamundo et al. suggested that the success rate is associated with longer fistulous tracks, but, in our study, there was no significant correlation between the length of the track and the recurrence rate [19]. Giamundo et al. reported an overall success rate of 33.3%, including 30 patients with transsphincteric fistulas [19]. Terzi et al., in a study of 103 patients, reported a disappointing 40% success rate that may be attributable to the fact that a seton was not used before the definitive treatment, nor was the internal opening closed [23]. They also described FILAC as a promising treatment option for anal fistulas with modest expectations and the possibility of reapplication.

Marref et al. argued that patients with high transsphincteric fistulas and suprasphincteric fistulas would benefit most from FILAC because of the advantage of preventing damage to the sphincter, unlike other conventional measures [24]. These researchers also suggested that patients with abscesses or infections should undergo drainage and placement of a loose seton to allow healing of the infection and convert it to a simple track with no cavity. Further, they suggested that the internal opening could be closed with a figure-eight Vicryl suture or left open, and the external opening left open or even excised for proper drainage. In our study, FILAC was combined with LIFT in four cases with high transsphincteric fistulas in order to achieve good results. Since FILAC is considered a blind technique, Marref et al. concluded that it could be combined with video-assisted anal fistula treatment (VAAFT) to overcome this potential shortcoming to make the side branches visible and that the combination of sphincter-saving procedures improved healing of fistulas and showed promise for the definitive management of complex perianal fistulas [24].

In our series, there was a significant correlation between the caliber of the track and recurrence, in that fistulas with a caliber of > 7 mm were more likely to recur. This finding is consistent with the suggestion of Giamundo et al. that the diameter of fistula tracts also deserves careful consideration. Despite the difference in the amount of energy delivered, the shrinking effect caused by the laser energy should allow fistulas with diameters not exceeding 4-5 mm to heal [25]. The fact that laser treatment may not be efficient for large-caliber fistulas could explain the higher failure rates in some series, for Giamundo et al. reported a failure rate of 21.7% and a recurrence rate of 11.4% over 10 years among 180 patients who underwent FILAC [25]. They also reported a statistically significant correlation between seton placement and recurrence.

Regarding the position of the fistulas, we observed a significant correlation between the posterior position and recurrence. Accordingly, we performed fistulotomies using a laser probe in three cases with posterior intersphincteric fistulas. No previous study has supported this approach; thus, Giamundo et al. included only anterior intersphincteric fistulas because most posterior intersphincteric fistulas are superficial, and fistulotomy is sufficient for their definitive treatment [25].

## Conclusions

The use of a diode laser is a safe and minimally invasive procedure for the treatment of hemorrhoids, chronic anal fissures, and perianal fistulas, being associated with minimal postoperative pain, short hospital stays, and rapid return to normal activities. The only limitation of the procedure is that it is expensive. We strongly recommend the use of a diode laser to treat these conditions, especially third-grade hemorrhoids and anterior single-branched fistulas with a caliber of < 7 mm in diameter, as a suitable alternative to conventional surgery.
